# Utilizing Sphingomyelinase Sensitizing Liposomes in Imaging Intestinal Inflammation in Dextran Sulfate Sodium-Induced Murine Colitis

**DOI:** 10.3390/biomedicines10020413

**Published:** 2022-02-09

**Authors:** Tuula Penate Medina, Jie Pan, Christabel Damoah, Jana Humbert, Anna-Lena Köpnick, Olga Will, Susanne Sebens, Oula Penate Medina

**Affiliations:** 1 Section Biomedical Imaging, Department of Radiology and Neuroradiology, University Hospital Schleswig-Holstein Campus Kiel, Kiel University, 24118 Kiel, Germany; Tuula.penate@rad.uni-kiel.de (T.P.M.); christie78040@gmail.com (C.D.); jana.humbert@rad.uni-kiel.de (J.H.); akoepnick@pharmazie.uni-kiel.de (A.-L.K.); olga.will@rad.uni-kiel.de (O.W.); 2 Institute for Experimental Cancer Research, Kiel University, 24105 Kiel, Germany; panj@zju.edu.cn (J.P.); susanne.sebens@email.uni-kiel.de (S.S.); 3 Department of Endocrinology and Metabolism, Second Affiliated Hospital of Zhejiang University School of Medicine, Hangzhou 310058, China; 4 Department of Radiology and Neuroradiology, University Hospital Schleswig-Holstein Campus Kiel, 24105 Kiel, Germany

**Keywords:** SM-liposomes, ICG, DSS, sphingomyelinase, targeting, optical imaging, macrophages, inflammation, photoacoustic imaging (PA)

## Abstract

Inflammatory bowel disease (IBD) is characterized by chronic inflammation in the gastrointestinal tract, resulting in severe symptoms. At the moment, the goal of medical treatments is to reduce inflammation. IBD is treated with systemic anti-inflammatory compounds, but they have serious side effects. The treatment that is most efficient and causes the fewest side effects would be the delivery of the drugs on the disease site. This study aimed to investigate the suitability of sphingomyelin (SM) containing liposomes to specifically target areas of inflammation in dextran sulfate sodium-induced murine colitis. Sphingomyelin is a substrate to the sphingomyelinase enzyme, which is only present outside cells in cell stress, like inflammation. When sphingomyelin consisting of liposomes is predisposed to the enzyme, it causes the weakening of the membrane structure. We demonstrated that SM-liposomes are efficiently taken up in intestinal macrophages, indicating their delivery potential. Furthermore, our studies showed that sphingomyelinase activity and release are increased in a dextran sulfate sodium-induced IBD mouse model. The enzyme appearance in IBD disease was also traced in intestine samples of the dextran sulfate sodium-treated mice and human tissue samples. The results from the IBD diseased animals, treated with fluorescently labeled SM-liposomes, demonstrated that the liposomes were taken up preferentially in the inflamed colon. This uptake efficiency correlated with sphingomyelinase activity.

## 1. Introduction

Both ulcerative colitis and Crohn’s disease, two primary forms of inflammatory bowel disease (IBD), are chronic conditions of gastrointestinal tract inflammation. An amount of 19.2 to 24.3 new cases for ulcerative colitis per 100,000 inhabitants are reported a year for Europe and North America. In contrast, Crohn’s disease has slightly lower incidence rates (12.7–20.2 cases per 100,000 inhabitants/year) [1]. The seriousness of the symptoms varies among patients, and the symptoms can be severe and may lead to life-threatening complications requiring surgical removal of the diseased tissue. Colitis is one of the conditions that also increases the risk of bowel tumor formation [2,3]. The treatment of IBD focuses on minimizing inflammatory responses and states with anti-inflammatory and immuno-modulating agents, combined with dietary and environmental adjustments [4,5]. Therapies include injectable anti-tumor necrosis factor-α (TNF-α) drugs that induce healing of the affected mucosa [6,7]. 

Liposomes and micelles are among the most common nanostructures used in clinical drug delivery applications [8]. Their use reduces the required systemic drug doses and minimizes the risks of adverse side effects. Liposomes can also be used for the solubilization of drugs, and they may prolong the half-life of the drug in the blood [9]. Once accumulated at the target site, the active agents carried by the liposomes have to be released. We have developed a liposomal system where the loaded material can be removed controllably [10,11]. We use sphingomyelin (SM) in the liposomal membrane in this setup. The endogenous disease secreted sphingomyelinase (SMase) can attack the lipid membrane and induce liposomal leakage. Ceramide, the product of SMase action, is well known for destabilizing membrane bilayer structures [12,13] and causes liposome leakage [14]. SMase, which hydrolyzes sphingomyelin, is involved and increased in several diseases and stress events [15,16]. Based on previous studies, elevated acid sphingomyelinase (aSMase) activity also plays a role in bowel inflammation [17,18]. Accordingly, the aSMase inhibitor SMA-7 has been shown to prevent the symptoms of dextran sulfate sodium (DSS) induced colitis in mice [18]. Moreover, inflammatory cytokines and lipopolysaccharides are also inducers of SMase secretion. Accordingly, our SM-liposomes were successfully tested earlier in inflammation conditions [19,20]. 

Several immune cells are active and abundant in inflammation conditions, and SMase or ceramide increase has a regulatory role in this scenario. In tumor inflammation, lipid-loaded macrophages have been shown to secrete aSMase [21]. Furthermore, aSMase mediates CD3 and CD28 signals and controls CD4+T-cell activation and helper T cell responses [22]. The absence of the enzyme in aSMase-deficient mouse causes alteration of T cells subpopulations, diverting the cells toward a pro-inflammatory phenotype [23]. In systemic lupus erythematosus (SLE), neutral SMase is down-regulated in B cells, and the dysfunction of SMase worsens the inflammatory response [24]. Liu et al. [24] have also shown that LPS treatment increases SMase activity in murine macrophage cell line RAW 264.7. In contrast, Rozenova et al. showed that aSMase has a role in TNF-α secretion through regulating the tumor necrosis factor-α converting enzyme (TACE) activity in macrophages after LPS mediated stimulation [25].

However, the role of aSMase in IBD is not well understood, including its impact on the differentiation of macrophages, one of the most abundant immune cell populations in IBD tissues. Macrophages are specialized cells with vital roles in phagocytosis, host defense, and wound healing, e.g., through the regulated expression of inflammatory mediators. Both activated macrophages and SMase, which they secrete into the surrounding tissues, would offer an excellent target for SM-liposome delivery. In general, two extreme phenotypes of macrophages can be differentiated, the activated (M1) pro-inflammatory and the alternative (M2) anti-inflammatory macrophages mirroring the dichotomy of T helper cells. The cell’s polarization is dependent on cytokine combinations given by the local microenvironment, and the IBD outcome is significantly reliant on the levels of cytokines in this milieu. In IBD, M1-macrophages are overactive, and a method to reduce the M1-activity is dearly needed. This study aimed to investigate the effects of SM-liposomes on macrophages. 

Thus, the overall aim of this study is to investigate whether SM-liposomes can be used for bowel inflammation imaging and targeting. Our SM-liposomes target aSMase and can therefore download their content directly in the near vicinity of cells with elevated aSMase activity. While new drug delivery methods would benefit IBD treatment, we wanted to test the feasibility of our SM-liposomes in IBD imaging and delivery. We analyzed uptake of SM-liposomes by different cell types in vitro. Furthermore, we studied the aSMase participation in IBD and the suitability of the SM-liposomes in vivo in an IBD mouse model. DSS is used to induce subacute inflammation in the gastrointestinal tract in this model. DSS is a negatively charged polysaccharide that causes epithelial cell damage and triggers dysregulated immune responses changing the mucosa barrier’s function [26]. Animals exposed to DSS have gross rectal bleeding with loose stool (diarrhea), fecal hemoccult, and weight loss [27,28]. These symptoms are used to evaluate the severity of colitis [28] in our animals. SM-liposomes were loaded with fluorescent cardio green (ICG) for liposomal tracking in vivo. Overall, the results indicate that SM-liposomes have an excellent potential for IBD treatment in the future. 

## 2. Materials and Methods

**Liposomal Preparations.** Lipids from Avanti Polar Lipids (Alabaster, AL, USA), ICG from Sigma (Germany), and carboxyl-coated 5 nm Fe3O4 nanoparticles from AC Diagnostics Inc. (Fayetteville, AR, USA) were used in the study. The liposomes were prepared as previously described [10]. A lipid mixture of DSPC/cholesterol/SM/DOTAP (20:30:30:20 mol%, respectively; a total of 20 µmoles of lipids) was used. Lipids were added to a round-bottomed glass tube, and the chloroform lipid mixture was dried under nitrogen and lyophilized for 24 h. As a result, a dry chloroform-free lipid layer formed on the glass surface. Lipids were hydrated for 30 min in 60 °C PBS containing ICG (1 mg/mL) and nanoparticle iron (at a final concentration of 0.3 mg/mL). The spontaneous liposomes were formed with the water. The unilamellar liposomes were created using the small-scale LIPEX^®^ Extruder (Northern Lipids, Burnaby, BC, Canada) with 100 nm pore size membranes (Sigma-Aldrich Chemie, Taufkirchen, Germany). PD-10 Sephadex column (GE Healthcare, Chicago, IL, USA) was used to purify the liposomes. The 1.5 mL fractions were collected, and the ICG fluorescence of the fractions was measured to estimate the encapsulation efficiency. The liposomal size was controlled using dynamic light scattering (DLS) and transmission electron microscopy (TEM). Liposomes were labeled with SYTO™RNASelect™green (Invitrogen, Bremen, Germany) for the cell studies. These were added to the lipid mixture and dried as described above.

**Macrophage isolation and used cell lines.** Monocytes were isolated from healthy blood donors by counterflow centrifugation, as described. For further experiments, only monocytes with a purity of a minimum of 90% were directly used or differentiated into M1- or M2-macrophages. Isolation of monocytes and their differentiation into macrophages and their phenotypic characterization were conducted according to established protocols revealing macrophage populations with high purity [29]. All donors gave their informed consent. Approval was obtained by the ethics committee of the Medical Faculty at Kiel University (Schleswig-Holstein, Germany) (reference number: D490/17). After differentiation, macrophages were seeded 100,000 cells/mL/well, and the cells were treated with 50 ng/mL TNF-α (Human recombinant, Sigma Aldrich, St. Louis, USA). The colorectal cancer cell line Caco-2(ATCC, HTB-37) was used as a model system of intestinal epithelial cells to compare liposomal uptake between inflammatory and epithelial cells. All the cells are cultured according to the ATCC guidelines. 

**Cell Studies.** Monocytes, different macrophage cell populations (M1 and M2 macrophages), and Caco-2 cells were seeded at 100,000 cells/mL/well a day before TNF-α treatment. The growth media was removed from all cells, and the cells were treated with 50 ng/mL TNF-α (Human recombinant, Sigma Aldrich, St. Louis, MI, USA) in PBS for 5 min, 120 min, and 360 min. Control cells were only treated with plain PBS. After treatment, the supernatant was collected, and secreted aSMase activity was measured.

In uptake studies, SM-liposomes loaded with RNASelect were used. After TNF-α treatment (360 min), the supernatant was removed from TNF-α treated and control cells, and cells were blocked with 1% bovine serum albumin (BSA) for 15 min. After incubation, BSA solution was removed, and 50 µL of liposomes (20 mM lipid mixture final dilution of 1:20) were added to the cells for 30 min at 37 °C. After the treatment, the cells were washed three times with PBS, and fluorescence intensities were measured using the Infinite^®^ M200 PRO plate reader (Tecan, Männedorf, Switzerland). Free RNASelect in PBS was given as a control for the liposomal delivery, and the cells without TNF-α treatment were used as a control for TNF-α treatment. The experiments were performed in triplicate.

**Animal Studies.** VFB mice (Source Charles River) were kept in temperature and humidity-controlled conditions with a 12 h light-dark cycle and free access to food and water. Mice were divided into two random groups: with a DSS treatment (*n* = 20) and control (*n* = 12) animals. The local animal experimentation ethics committee approved the experiments (MELUR, license number V 312-7224.121-17 (74-6/13), and they were carried out following the guidelines for animal care at the University of Kiel. 

DSS was used to create an acute colitis mouse model. The model was established according to the protocols described previously [23,30]. In this study, DSS (45 kDa; TdB Consultancy, Sweden) was added to drinking water at a concentration of 4% for five days. The treatment was changed on the sixth day to regular water. The inflammation status and progression were monitored with the disease activity index (DAI) score [31]. The scores of each DAI parameter were taken for each mouse daily.

**Imaging studies.** The ICG-loaded liposomes were applied via tail vein injection (125 µL/animal). Before imaging, animals were anesthetized with an intraperitoneal injection (125 µL cocktail combination of 0.6% ketamine (AVECO Pharmaceuticals, Boston, MA, USA) and 0.4% medetomidine (Pfizer Deutschland, Berlin, Germany), and 4.0% NaCl). The animals were sacrificed 24 h after liposome injection, and the intestines were collected and imaged ex vivo with the NightOWL 983 (Berthold Technologies, Bad Wildbad, Germany). The imaging was performed with excitation/emission filters of 740 nm/790 nm for ICG, and images were created and analyzed with the indiGO software. Regions of Interest (ROIs) were drawn around two colon segments (proximal and distal colons). To further verify the targeting results obtained from the optical imaging using the NightOW, the OdysseyCLx was used. Tissue slices of 10 µm were cut, and ICG fluorescence was analyzed from non-stained histological samples with the Odyssey^®^CLx (LI-COR, NE, USA). These images were analyzed using ImageJ software. 

**Histological studies.** Clinical material and data were obtained from the clinical research database of the biobank BMB-CCC. Patients who donated residual colonic tissue samples resected by routine surgery to the Comprehensive Cancer Center Kiel (BMB-CCC) gave informed consent to participate in this study. The study was approved by the local ethics committee of the Medical Faculty, Kiel University, and the University Hospital Schleswig-Holstein, Campus Kiel (Reference No. A110/99). 

Collected proximal and distal colon samples were embedded in paraffin, and 3 µm and 10 µm slices (the latter for ICG fluorescence analysis) were cut. The slides were deparaffinized and then demasked by cooking in a vacuum with citrate buffer pH 6.0 and blocked with peroxidase block, followed by blocking with 10% nonfat dry milk. The sections were stained with AEC (Zytomed, Bargteheide, Germany) and counterstained with hemalum solution (Waldeck, Münster, Germany). For immunohistochemistry (IHC) analysis, the sections were stained with a primary antibody specific for aSMase (bs-6318R, Bioss Antibodies Inc., Woburn, MA, USA) or macrophages (CD68; MyBioSource, San Diego, CA, USA) and diluted 1/1000 in 10% milk, followed by HRP-polymer-anti-Rabbit (Zytomed, Bargteheide, Germany). Universal negative serum (Biocare, Metten, Germany) was used for the negative control staining. The slides were attached with Pertex and counterstained with hemalum. Quantification was done by measuring the area in crypt folds positive for aSMase staining and referring the resulting values to the total crypt fold area. Bars represent mean ± SEM. *n* = 3 (three mice per condition).

**Acid sphingomyelinase activity assay.** aSMase activity was analyzed from collected cell supernatants or colon tissue homogenates using an Amplex Red reagent-based fluorometric kit (Thermo Fisher Scientific, Waltham, MA, USA) as previously described [10]. The tissue samples were weighed and homogenized. The fluorescence caused by sphingomyelinase activity was analyzed from the homogenate or cell supernatant. The assay was performed in acidic conditions. For comparison, in the tissue experiment, the aSMase activity was also analyzed from colons gathered from healthy animals.

**Statistical analyses.** Statistical analysis from cell and animal experiments was done using PRISM software from GraphPad. All significant differences displayed in the figures are calculated using an unpaired *t*-test.

**Photoacoustic Imaging.** Photoacoustic imaging was done with a custom-built real-time optoacoustic imaging system photoacoustic device (MSOT inVision 256-TF, iThera Medical, Munich, Germany) using an external 3D probe which has a three-dimensional array of 384 spherically focused transducer elements, each with a center frequency of 2.5 MHz (45% bandwidth, 4 cm radius of curvature, and maximal spatial resolution of 310 µm). The ICG loaded SM-liposomes were injected via the tail vein to a DSS-treated anesthetized animal (*n* = 3). The photoacoustic signal of ICG was traced 24 h later and analyzed as described earlier (Humbert, J. et al., 2020). 

## 3. Results

### 3.1. In Vitro Cell Studies

**Figure 1:**
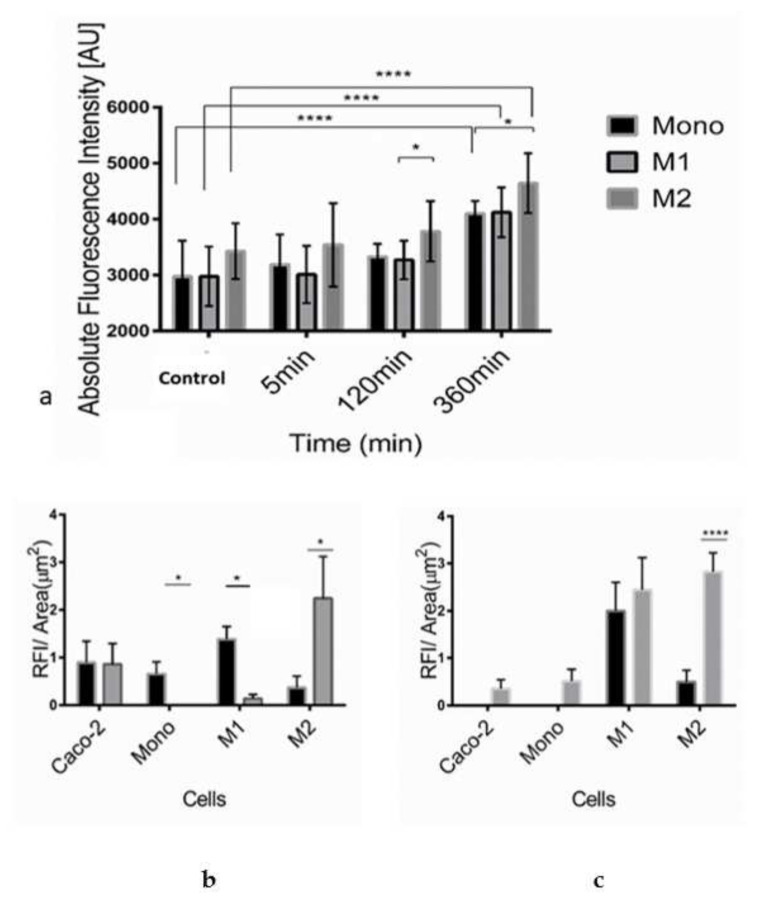
Acid SMase secretion was analyzed from the supernatants of human monocytes, M1- and M2-macrophages in the absence of TNF-α treatment (control) and after 5 min, 120 min, or 360 min treatment with 50 ng/mL TNF-α (**a**). Acid sphingomyelinase activity was studied using Amplex Red sphingomyelinase assay. In the assay, the formed ceramide is traced from the formed fluorescent by-product, which equals the formed ceramide’s molarity. Bars represent mean ± SEM of triplicate repeats (*n* =3). A non-parametric *t*-test determined significance. Liposomal internalization efficacy of monocytes, M1- and M2-macrophages, and Caco-2 cells were analyzed, measuring RNASelect fluorescence from the cells. Cells were incubated with SM-liposomes loaded with RNASelect (grey columns) or as control free RNASelect (black columns) in the absence (**b**) or presence (3 h) of 50 ng/mL TNF-α (**c**) for 30 min following washes with PBS for three times. Bars represent mean ± SEM of triplicate repeats. A non-parametric *t*-test determined significance. * *p* < 0.05, **** *p* < 0.0001.

The increased blood circulation in the inflammation area facilitates liposomal delivery to the site. We have previously shown that SM-liposomes target those sites of inflammation [19,20]. Still, it is not fully understood which cells take up liposomes and the material loaded in the inflammatory microenvironment. For this purpose, we studied the fate of liposomes in the interaction with monocytes and macrophages exhibiting different phenotypes. In addition, we also analyzed liposomal uptake in the colon cancer cell line Caco-2, which is commonly used as a model of intestinal epithelial cells. Furthermore, aSMase secretion was studied in a simplified inflammatory environment created by adding TNF-α to the cell medium, as TNF-α is known to be increased in IBD [32]. We could demonstrate that the secretion of aSMase by macrophages depends on their differentiation into an M1- and M2-phenotype showing the highest secretion by the M2-subtype compared to monocytes and M1-macrophages (Figure 1a). The secretion increased significantly during TNF-α exposure in all three monocytic cell populations, being the highest in M2-macrophages after 360 min of exposure. It is known that biological aSMase reactions are rapid, which is why we focused on studying early time points. 

Interestingly, when aSMase secretion of macrophages was compared with the secretion of Caco-2 cells, TNF-α treatment affected only the increase in macrophages (Table 1).

In liposomal uptake studies, Caco-2 cells were used in addition to macrophages. The cells were treated with SM-liposomes loaded with RNASelect, a marker for RNA delivery. It is a dye that fluoresces only when bound to RNA [33]. In normal cellular uptake, the RNASelect can be taken in the cells, and a small portion can find their way to the cytosol and thus contact the RNA. However, in the case of the liposomal nanoparticles, the liposome hinders the RNAselects access to the cell. Only when there is an active intake or liposome rupture may the RNASelect reach the cytosol. Surprisingly after TNF-α treatment, more liposomal RNASelect is found on the cytosol than the free RNASelect group. 

The internalization of the liposomes was analyzed in the absence (Figure 1b) or presence of 50 ng/mL TNF-α for 3 h (Figure 1c). Free RNASelect was added to the cells to control the free diffusion of the molecules to the cells. As demonstrated in Figure 1b, it seemed that there is some free diffusion of free RNASelect to, mainly, Caco-2 cells and M1-macrophages.

The uptake of RNASelect is dependent on the formulation. The results indicate a decrease in the liposomal uptake of RNASelect to Caco-2 cells after TNF-α treatment (Figure 1c). A similar but milder pattern could be seen for the uptake of free RNASelect of Caco-2 cells. The uptake was decreased in Caco-2 cells after TNF-α treatment but stayed stable in M1-macrophages. Monocytes do not internalize liposomal RNASelect; however, free RNASelect as a small molecule can moderately cross the plasma membrane even without TNF-α treatment. A similar significant pattern can be seen in the M1 macrophage population. M2 macrophages take significantly more liposomal RNASelect than free RNASelect without TNF-α. The liposomal delivery was particularly efficient in Caco-2 cells and M2-macrophages. However, the phagocytic potential altered significantly in M1-macrophages, showing a roughly tenfold increase in the SM-liposome uptake after TNF-α stimulation. In TNF-α treated M1-macrophages, an apparent increase could be observed in liposomal uptake. In contrast, M2-macrophages exhibited a high steady-state of internalization before and after TNF-α treatment. The phagocytic potential and SMase secretion provide an excellent opportunity for our liposomal targeting. Interestingly, compared to the epithelial-derived Caco-2 cells, the liposomal delivery potential was remarkably better in macrophages. Overall, these intake results open the possibility for macrophage-specific liposomal targeting.

### 3.2. aSMase Analysis in DSS Murine Colitis Model and Human Tissue Samples

**Figure 2:**
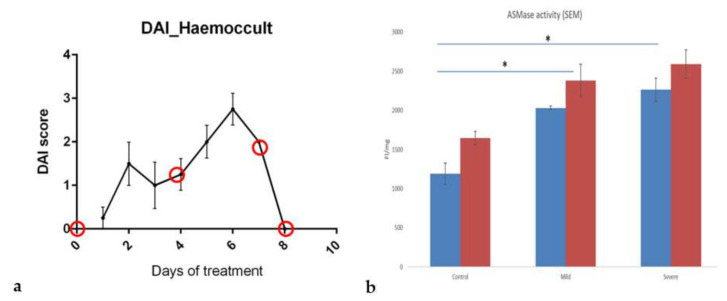
The inflammation status and progression of the disease were tracked with the disease activity index (DAI) score in DSS-treated mice (**a**). The DAI combines weight loss scores to initial body weight, stool consistency (fecal score), and hemoccult (fecal bleeding). The scoring system can be employed for the comparative analysis of intestinal bleeding [28]. The red circles present the chosen endpoints where animal colons were collected (*n* = 4 in each endpoint) and analyzed. Acid sphingomyelinase (aSMase) activity was measured from the mouse proximal (blue column) and distal (red column) colon homogenates using Amplex red aSMase assay kit (**b**). The tissue samples were collected from healthy, mildly inflamed (three day DSS treated) and severely inflamed (five day DSS treated) animals (*n* = 4 in each group). The samples were weighed and homogenized, and fluorescence was analyzed. The fluorescence intensity (FI) was standardized with the tissue weight (mg). The animals without DSS treatment were used as the control. All tissue sample activities were analyzed simultaneously, and data are presented as mean ± SEM. A *t*-test determined significance. * *p* < 0.05.

Some previous studies have already demonstrated the relevance of SMase or an increase in sphingolipid metabolism in IBD [17,18,34]. We used the DSS-induced colitis mouse model to substantiate that SM-liposomes are suitable delivery tools in IBD. In this model, we focussed on the sub-acute phase of the disease because early detection and treatment initiation would be the most desirable for those patients. DSS (45 kDa) was added to drinking water at a concentration of 4% for five days to induce colitis of the epithelial cells of the basal crypts of the intestines. The treatment was changed on the sixth day to regular water. Figure 2a represents the disease activity index (DAI) in the treated animals demonstrating a constant rising of DAI upon DSS-treatment from day three to day six. 

Next, aSMase activity levels were analyzed in the diseased colon. For this purpose, tissue samples were collected from animals treated with DSS for three days (corresponding to a mild inflammation with a DAI score of 1 at day three, or 2 at day five; corresponding to a severely inflamed tissue with a DAI score of 2–8) and non-treated control animals. Enzyme activity was determined in tissue homogenates from proximal and distal parts of the colon using the Amplex red aSMase assay. Compared to the healthy tissue samples, aSMase activity was clearly and significantly increased in mildly inflamed tissues and even more in tissues with severe inflammation (Figure 2b). Moreover, the enzymatic activity was higher in all healthy and diseased tissues in the distal part than in the colon’s proximal part.

**Figure 3:**
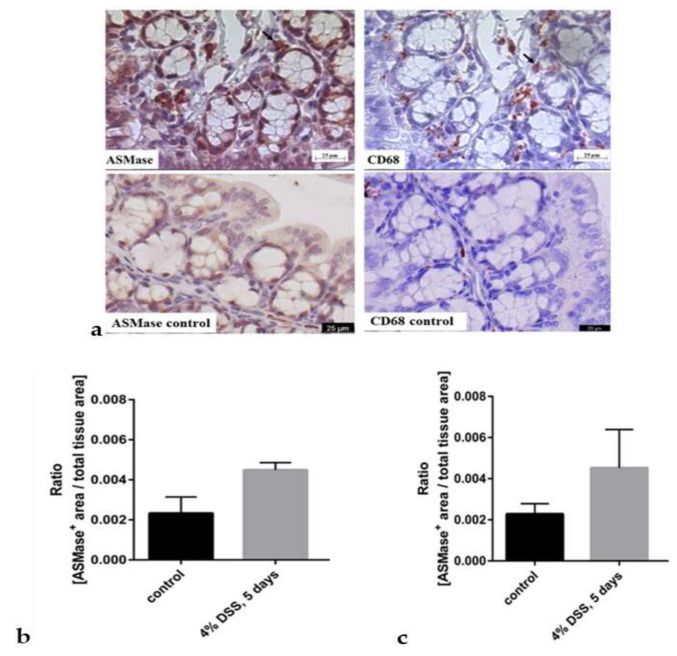

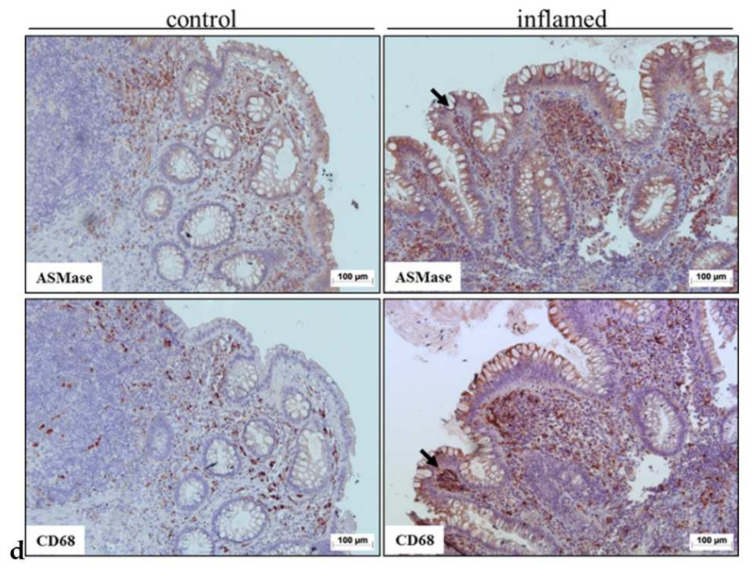
Immunohistochemical staining of aSMase (left upper panel) and macrophages (CD68) (right upper panel) in colons of mice suffering from dextran sodium sulfate (DSS)-induced acute colitis (upper row) (**a**). Mice were sacrificed after receiving 4% DSS for five days in their drinking water to induce acute colitis. The control animals had plain water (lower raw), mice were sacrificed, and colons were collected for immunohistochemical aSMase staining. Representative images of the same crypt fold of the proximal colon are shown. Arrows indicate possible co-localization. Scale bar = 25 µm. The lower row presents representative proximal colon from healthy control animals stained with aSMase (left) and CD68 (right).The aSMase expression was quantified from the histological slides (**b**). Quantification was done by measuring the area in crypt folds positive for aSMase staining and referring the resulting values to the total crypt fold area in the proximal colon (**b**) and distal colon (**c**). Bars represent mean ± SEM. (*n* = 3 per condition). Sphingomyelinase appearance was also analyzed from a human patient sample (**d**). Immunohistochemical staining of aSMase (upper row) and macrophages (CD68) (lower row) in colon tissue from a patient who has Crohn’s disease. Representative images show areas covering several crypts and crypt folds. Colonic tissue from areas regarded as non-inflamed (left) and inflamed (right) was collected, sliced, and immunohistochemically stained. Scale bar = 100 µm.

Next, we wanted to verify further the enzyme’s appearance in the inflamed tissue using immunohistochemical stainings of aSMase. Quantification of aSMase expression from stained control and inflamed colons revealed that aSMase expression was elevated in the DSS-treated colon tissues, the proximal colon (Figure 3b), and the distal colon (Figure 3c) samples. Thus, the aSMase staining confirmed the results of the elevated aSMase activity in inflamed colon tissues (Figure 2b) compared to the colons of healthy animals. Based on our findings of much aSMase activity in macrophages (Figure 1a), we next investigated whether aSMase expression is associated with the abundance of macrophages. Since aSMase activity was increased in the presence of both macrophage subtypes in our in vitro studies, we stained the colon tissues of healthy and DSS-treated animals for the general macrophage marker CD68 and aSMase antibody. There was an apparent increase in aSMase staining in inflamed slices compared to non-inflammatory control slices. Immunohistochemical analysis revealed that aSMase is found in some cases at macrophages in the colon tissue samples (Figure 3a,d). There are also aSMase stained epithelial cells around the crypts in the inflamed slides. While there are more epithelial cells than macrophages, it seems that from the overall inflamed tissue, aSMase increase is due more from epithelial aSMase.

CD 68 staining shows that there are more macrophages in the inflamed tissue than the non-inflamed tissue. Also, elevated aSMase expression and activity might be related to the high abundance of macrophages in the inflamed colon tissues. 

To further substantiate the link between colonic inflammation and increased aSMase expression, aSMase was stained in surgically resected colon tissues of IBD patients. As the murine tissues, human IBD tissues were stained immunohistochemically with an aSMase antibody (Figure 3d, upper raw) and a macrophage-specific CD68 antibody (Figure 3d, lower row). Similar to our findings from the colon tissues of the DSS-treated mice, we could detect aSMase expression in the human tissues being found in similar regions as the macrophage marker CD68.

### 3.3. Liposomal Targeting In Vivo

**Figure 4:**
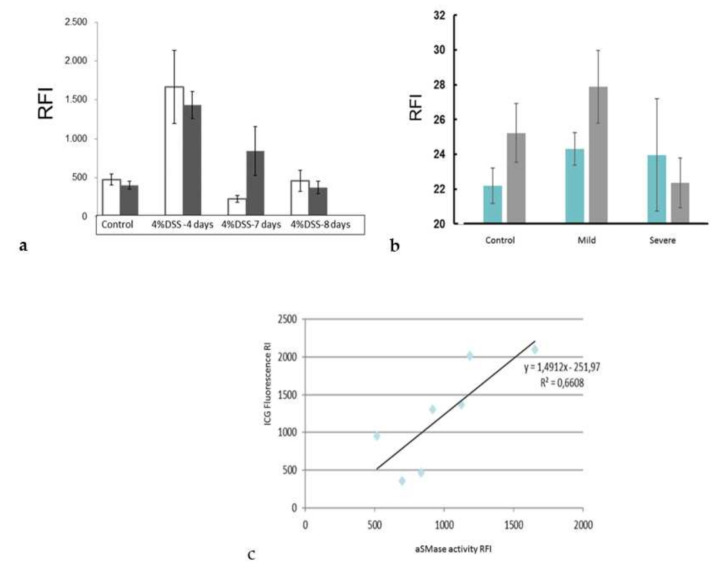
The inflammation targeting of the ICG loaded SM-liposomes was studied ex vivo from dextran sodium sulfate (DSS)-treated animals (**a**). The mice were treated with 4% DSS in drinking water for five days, and then regular water was given until the endpoint. Animals were sacrificed on days four, seven, and eight. Healthy non-treated animals were used as a control. The liposomes (125 μL 20 mM total lipid in PBS) were added via tail vein injection to the animals (n = 4 per disease group and control group). The mice were sacrificed, and the ICG fluorescence was analyzed from the proximal colon (dark column) and distal colon (white column) ex vivo 24 h after tail vein injection using a Night Owl Imager. Regions of Interest (ROIs) were drawn around two colon segments (proximal and distal colons) and were analyzed with the indiGO software. Y-axes represent Relative Fluorescence Intensity (RFI). Bars represent mean ± SEM. The targeting of the ICG- loaded SM-liposomes was verified by analyzing the ICG fluorescence from histological slices (**b**). The proximal (blue) and distal (grey) colon samples were collected and cut into 10 μm thin section slices. The fluorometric scanner was used to measure the ICG fluorescence from the thin section slices. Bars represent mean ± STDEV. Correlation between the aSMase activity (*x*-axel) and targeted ICG fluorescence (y-axel) was analyzed (**c**). The ICG fluorescence was measured from proximal colon samples using a Night owl Imager; ROIs were drawn around proximal colon samples and were analyzed with the indiGO software. The homogenate samples were collected from the same proximal colon samples, and aSMase activity was analyzed using an Amplex Red acid sphingomyelinase assay kit. The Amplex fluorescence was standardized to the tissue weight (mg). All the tissue sample activities were analyzed simultaneously.

After demonstrating the presence of aSMase in the inflamed colon tissues, we wanted to test whether our SM-liposomes can target the site of inflammation. For this purpose, SM-liposomes were loaded with ICG. Labelled liposomes were added via tail vein injection, and 24 h later, animals were sacrificed to collect colons from diseased and healthy mice to trace the liposomes. Colon samples were imaged, and the ICG fluorescence was analyzed. Liposomal targeting was most effective in the early phases of inflammation (day four), and a decline is observed at later time points when DSS was removed. The DAI score was declined to baseline levels (Figure 4a). Moreover, targeting was higher in diseased colons compared to colons from healthy animals (Figure 4a). On day four, the time point with the most increased liposomal targeting, both distal and proximal colon tissues demonstrated improved liposome targeting. We generated slices from diseased (five days, 4% DSS) and healthy colons to verify the result and traced the ICG fluorescence from the tissue slices using a fluorescence scanner. As demonstrated, the thin section slices gave similar results to the *ex-vivo* intestine analysis (Figure 4b). However, this analysis lacks sensitivity compared to topical fluorescence imaging (Figure 4a). Interestingly, aSMase appearance and targeted ICG were highest on day four of DSS treatment, as shown in Figure 4c.

**Figure 5:**
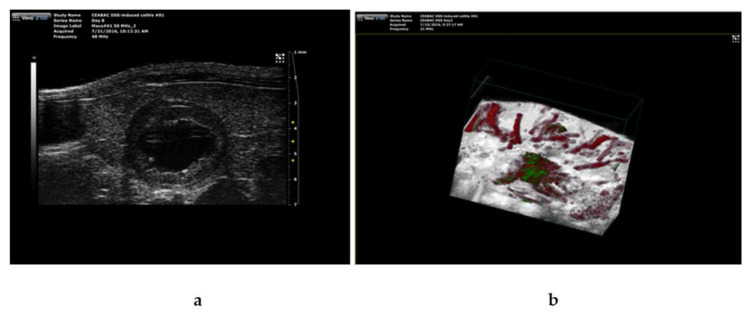
Photoacoustic imaging combined with ultrasound imaging was used to verify the severity of inflammation and ICG targeting to the site of inflammation in DSS-treated mice. The mice ( *n* =3) were treated with 4% dextran sodium sulfate (DSS) for four days before the addition of ICG-loaded SM-liposomes via tail vein injection (125 μL 20 mM total lipid in PBS). A representative in vivo image from the colon of the DSS treated mouse using ultrasound (**a**) and photoacoustic visual, sonic device (**b**) are shown. ICG signal detected by spectral unmixing is presented in green, and red represents the hemoglobin signal in the PA image.

As we have previously shown that ICG-loaded SM- liposomes are excellent tracers in photoacoustic (PA) technics [35,36,37], we used PA imaging to validate ICG liposome targeting the inflamed tissues in the DSS treated mice. Figure 5 demonstrates a representative image of a colon of mice at day four of DSS treatment with ICG liposomes applied 24 h earlier (Figure 5b). Confirming our results from ex vivo imaging (Figure 4a), ICG localization in the inflamed tissue could be observed. Furthermore, ultrasound examination combined with photoacoustic imaging confirmed the inflamed condition of the colon, showing the swelled inner part of the colon (Figure 5a). 

## 4. Discussion

Inflammation treatment in IBD would benefit immune cell-specific targeting. Once the liposomes have accumulated at the target site, the active agents carried by the liposomes have to be released, preferably in an efficient and controllable manner. When sphingomyelin is included to the liposomal membrane, endogenous disease secreted sphingomyelinase can attack the membrane, cut sphingomyelin, and induce liposome leakage. SM- lipid-containing liposomes have been used successfully for years in clinical formulations of anticancer drugs [38]. Several studies already exist in the cancer field that could be implemented to chronic inflammatory diseases as well. Some findings show that SM-liposomes have superior release and circulation properties compared to other phospholipid liposomes [39]. The recent reports that ceramide homeostasis regulates macrophage activity and that liposomes can affect macrophages [19,40,41] suggest that liposomes could be a valuable tool to treat chronic inflammation and manipulate the macrophage subset balances. SMase secretion from macrophages has been shown earlier by other researchers [11,22,23], but, also, the variation in secretion between the subtypes is essential to understand. The difference in secretion behavior or liposome intake offers a possibility to microenvironment-dependent targeting. We analyzed aSMase secretion of different macrophage subsets after TNF-α stimulation a. We demonstrated that the secreted aSMase activity varies among monocytes and different macrophage subpopulations. In the pro-inflammatory M1-population, an elevation in aSMase secretion was detected, while anti-inflammatory M2-macrophages showed significant elevation of the aSMase. Although the M1 sub-population is slightly lower, the MI and M2 cells do not differ significantly in SMase secretion during the relatively short, 360 min, TNF-α activation period. If we look at the change in terms of percentage, there is very little difference. The TNF-α caused activation in aSMase secretion shows, after a bit of lag time, a linear increase in both M1 and M2 cell lines over the period, and this is significant. This is essential, while the aSMase can affect the M1/M2 specialization. Recent results from Roux-Biejat et al. (2021) show that aSMase regulates the balance between M1 and M2 macrophages in the injured muscles so that the absence of the enzyme reduces inflammation [42]. This is caused by the altered polarization of M1 macrophages towards an M2 phenotype. On the other hand, aSMase favors M1 cells in some inflammation conditions, but some M2 sub-populations cells have aSMase inhibitory effect through VEGF [43,44].

What is also remarkable is that in this early time scale (360 min), there is no increase in the epithelial cell aSMase secretion. This difference is highly significant compared to monocyte cell lines. In the future, it would be interesting to see the VEGF expression after TNF-α stimuli in M1 and M2 macrophage populations. 

Interestingly, it has been recently shown that activated M1-macrophages can be inhibited and reprogrammed by ceramide-dependent mechanisms [24]. It is also essential to know where the payload ends up. Rajan et al. (2018) have shown that PEGylated liposomes suppress anti-tumor immunity [45]. Still, our preliminary results with the non-PEGylated SM-liposomes show that immune cells can also react to the liposomes and liposome intake based on the M1- or M2-state. The change we saw in uptake efficacy in M1-macrophages is interesting because the IBD disease environment seems to offer increased uptake ability for M1-macrophage targeted delivery of our liposomes. While there are several candidate cells for SM-targeting, the critical question is to verify the actual target cells. Once fully characterized, this opens new ways of precisely targeted delivery that could be used in several disease conditions, like in immune therapy.

A holistic understanding of the different cell lines like immune cells, endothelial cells, and epithelial cells all together in the tissue in the different faces of the inflammation is dearly needed. In our study, we wanted to concentrate on the early effect of the TNF-α exposure to the macrophages and compare this to epithelial cells. In acute inflammation situations, macrophages face this when they invade the disease site. The epithelial cells are used as a control as they are prevalent cells at the intestine inflammation site. The researchers in the publication of Miyake et al., (2016) show that TNF-α and IL-6 have a profound effect on the intake of mannitol of epithelial cells when they are administered over a number of days [46]. In our studies, in 360 min of exposure to TNF-α we do not see a significant uptake increase in the Caco-2 cells, but we do see a remarkable increase in the M1 macrophages. 

For delivery purposes, it is crucial to know how much liposome content is leaked in the interstitial cell compartment versus being internalized by cells. We used RNASelect as a test molecule for delivery. This was chosen because RNASelect gives its fluorescence when it binds specifically to RNA. The signal fluorescence only in the case of RNA-specific binding offers us an opportunity to test whether can we deliver the liposomal material to the cytosol of the cells. Successful RNASelect delivery also gives a possible indication that the delivery system could be used for RNA delivery. Our studies with RNASelect show that RNA delivery on site would also be a suitable treatment option in the future. However, this requires further investigation in the future. In this study, we could demonstrate that our liposomes can be delivered to the site of inflammation in a well-characterized model of DSS-induced acute murine colitis. The high occurrence of the active aSMase enzyme in diseased mouse colon tissues compared to healthy control ones shows the increased activity of the enzyme in diseased tissue. It provides the basis for the improved delivery of SM-liposomes into the disease site. We even could see a positive correlation between aSMase activity and delivered fluorescent ICG in the colon, indicating the suitability of SM-liposomes as a diagnostic agent.

Furthermore, we have demonstrated enhanced aSMase expression in IBD tissues. The enzyme expression was slightly higher in the distal colon than in the proximal colon in Figure 2b, where aSMase activity was presented per/mg of colon tissue. However, in evaluating aSMase from histological samples (Figure 3b,c), the difference was not clear between the distal and proximal colon and healthy tissue. Distal colon tissue was more damaged and more difficult to analyzes, which can be seen in the higher error bars in the diseased distal colon. Five-day treated animals are the most severe ones in our study, and based on the previous studies, it is known that the inflammation is most robust in the distal part of the colon. From histological samples, we could see that both in murine DSS-treated colon tissues and human IBD tissue specimens, aSMase expression is elevated. We can see the difference in the early phase of the disease in the proximal and the distal parts of the colon. Overall, these data indicate that aSMase activity is increased in inflammation-affected intestinal tissues. Moreover, a co-localization of macrophages and aSMase was observed in inflamed colon tissues. There was also strong aSMase staining in the epithelial cells of the crypts. Our observation fits well with the previous study of Sakata et al. [18], where the protective effect of aSMase-inhibition was mediated by suppressing cytokine production from macrophages in murine DSS-induced colitis. The presented findings suggest the suitability of SM-liposomes for macrophage targeting in IBD and that targeted delivery is already the strongest (and possible) treatment, even at the early phases of the disease.

IBD inflammation targeting would offer a convenient new treatment vehicle in the cause of the disease, where tissue damages are not yet that severe. Furthermore, the imaging point of view of early phase targeting might improve disease diagnostics. ICG as a tracer molecule offers several benefits in imaging. First of all, it is already a clinically approved marker molecule and is known to be safe in use. There is new imaging technology available with filters that are suitable for ICG. Thus liposomal targeting could also be used as a part of surgical operations in the future. ICG is also a photoacoustic marker molecule, so PA solutions would also be possible in future imaging. Photoacoustic methods are interesting because MRI imaging of the colon is challenging, while gas in the intestines gives an uneven background to the luminal side of the colon. PET alone is morphologically challenging in small animals, and PET-CT is not fully useful when imaging soft tissues. The photoacoustic technique is not a fully developed technique, but it allows excellent morphological imaging and a non-toxic optical tracer for enzyme-based molecular imaging.

Finally, an inflammation-targeted delivery system can deliver a wide variety of anti-inflammatory drugs and allow more nuanced drug treatment possibilities in the future. 

## 5. Conclusions

In this study, we confirmed that SM-liposomes could target the site of IBD inflammation. Our studies indicated that sphingomyelinase activity is increased in DSS-caused murine colitis and that the liposomes were taken up preferentially in the inflamed colon. The liposomal delivery correlated with the sphingomyelinase activity found in the diseased colon. At the cellular level, we demonstrated that SM-liposomes are efficiently taken up in intestinal macrophages. This is remarkably enhanced in M1 macrophages with TNF-α treatment.

## Figures and Tables

**Table 1:** Change of ASMase activity (U/mL) in cell supernatant after 120 min and 360 min treatment of 50 ng/mL TNF-α.

	TNF-α 120 min	TNF-α 360 min
**Caco-2**	99%	99%
**Monocytes**	111%	131%
**M1**	109%	140%
**M2**	107%	134%

Acid sphingomyelinase activity was measured using the Amplex red sphingomyelinase assay kit. Each measurement at every time point was done as a triplicate and repeated three times. The data are presented as a % increase of aSMase activity compared to untreated.

## Data Availability

All of the data is available upon request.
